# Influence of spine biomechanics and sagittal balance on the outcome of lumbar discectomy

**DOI:** 10.3389/fsurg.2025.1494780

**Published:** 2025-02-12

**Authors:** José Poblete Carrizo, Jesús Martínez, Julio González, Alejandra Mosteiro, Ramon Torné, Alberto Di Somma, José Ríos, Joaquim Enseñat, Salvador Fuster

**Affiliations:** ^1^ Department of Neurosurgery, Hospital Clínic de Barcelona, Barcelona, Spain; ^2^ Faculty of Medicine, University of Barcelona, Barcelona, Spain; ^3^ Department of Neurosurgery, Centro Médico ABC Santa Fe, Ciudad de Mexico, Mexico; ^4^ Department of Neurosurgery, Clínica RedSalud Providencia, Universidad de Santiago de Chile, Santiago de Chile, Chile; ^5^ Department of Clinical Pharmacology, Hospital Clínic de Barcelona, Barcelona, Spain; ^6^ Department of Orthopaedics and Traumatology, Hospital Clínic de Barcelona, Barcelona, Spain

**Keywords:** lumbar disc herniation, sagittal balance, biomechanic, microdiscectomy, prognosis, recurrence

## Abstract

**Purpose:**

Spine biomechanics, particularly sagittal balance and spino-pelvic angulation are determinant factors in the understanding of lumbar degenerative disease. These concepts translated into objective measurements are progressively being integrated into clinical practice. The present study explores them as prognostic factors in patients undergoing lumbar microdiscectomy, which could help identify those at higher risk of surgical failure.

**Methods:**

Prospective analysis of patients treated with lumbar microdiscectomy (*n* = 52) and healthy control subjects (*n* = 45) in a single tertiary centre. Follow up of 12 and 24 months after surgery, with radicular and lumbar pain evaluation according to the Visual Analogue Scale (VAS) and Oswestry Disability Index (ODI). Comparison of several objective spinal biomechanic factors, measured by a single experienced radiologist. Assessment of spinal sagittal balance as a prognostic factor after lumbar discectomy.

**Results:**

Compared to healthy individuals, patients with symptomatic lumbar disc herniation showed lower thoracic kyphosis (39.03 vs. 34.42° *p* = 0.034), lower thoraco-lumbar transition T10-L2 angulation (6.79 vs. 2.08° *p* = 0.005), lower lumbar lordosis (59.54 vs. 48.36° *p* < 0.001) and lumbo-sacral angulation L4-S1 (40.20 vs. 29.16° *p* < 0.001), lower pelvic incidence (54.71vs 49.86° *p* = 0.014) and lower sacral slope (42.07 vs. 33.34° *p* < 0.001). Sagittal balance (SVA) was negative in healthy subjects −3.09 mm and positive lumbar-disc patients 15.04 (*p* = 0.013). Noteworthy, the radicular and lumbar pain and disability outcomes 12 and 24 months after surgery were significantly better in the group with normal sagittal balance (ODI 14.52 vs. 40.06 *p* < 0.001; radicular VAS 2.74 vs. 5.58 *p* < 0.001; lumbar VAS 3.61 vs. 4.06 *p* < 0.001).

**Conclusion:**

Lumbar degenerative disc disease represents a major burden for healthcare systems; thus, its management is determinant. Lumbar discectomy shows overall positive results, with a significant reduction of pain and disability in the majority of cases. However, a subgroup of patients, still not well defined, may experience persistent pain after the intervention. The use of objective measurements of sagittal balance may help identify these patients for which simple discectomy may not suffice and contribute to treatment planification.

## 1 Introduction

The biomechanics of spine, particularly the degree of angulation in its sagittal plane and its relation to the pelvis, are determinant factors in the static balance and in the resistance to intrinsic and extrinsic axial load compression (1, 2). Therefore, the accurate definition of sagittal balance and its variations have gained importance in the understanding of the lumbar degenerative disease, its pathogenesis, evolution and prognosis after a surgical treatment (3, 4).

The sagittal balance can be evaluated through metrical and angular parameters. However, multiple systems have been proposed without a definitive uniform consensus. This results from the great interest that this matter raises and its neurosurgical and orthopaedic importance; yet, on the other hand, it is also a reflection of its complexity (5). Ultimately, the lack of an objective, precise and personalised definition of the spinal alignment limits its use in clinical practice and surgical decision making.

The spinopelvic parameters are paramount to evaluate the sagittal balance and to understand its influence in the lumbar disease. Duval-Beaupere et al. established specific measurements including the sagittal vertical axis (SVA), pelvic tilting (PT), the sacral slope (SS) and the lumbar lordosis (LL) (6). Roussouly et al. went on to classify the lumbar spine into five types, according to its sagittal plane (7).

These concepts are progressively being more integrated into clinical practice in an effort to extrapolate their anatomical importance to the management of several degenerative diseases, including lumbar disc. The present study will analyse the sagittal balance parameters and their relation to the clinical and functional outcomes of patients undergoing lumbar microdiscectomy. The primary objective is to determine whether a sagittal disbalance could be a predictive factor of poor outcome in these patients, something that could have relevant implications in the surgical management of this disease.

## 2 Methods

### 2.1 Study design

The study design has been approved by the institutional Ethics Committee (HCB/2021/0703) and complies with the Helsinki Declaration and national laws regarding biomedical research and treatment of personal data. It is a prospective case-control analysis, with the primary aim to compare the sagittal balance parameters with the development and clinical outcomes of patients undergoing microdiscectomy for lumbar disc herniation.

### 2.2 Population

Patients were sequentially recruited from the Spine Clinic of a single tertiary hospital of reference for lumbar degenerative disease, between January 2018 and December 2022. Inclusion criteria were: (1) Age 18–60 years, (2) diagnosis of lumbar disc herniation, (3) surgical indication for single-level lumbar discectomy. Exclusion criteria were: (1) Patients who had previous lumbar surgery, (2) patients undergoing additional arthrodesis, (3) patients with spondylolisthesis with Meyerding >1; (4) patients lacking the necessary imaging techniques for the aims of the present analysis, (5) patients who did not complete the two-year follow up, (6) patients with cauda equina syndrome requiring urgent treatment, (7) patients who had hip surgery or suffered from untreated hip deformities. Diagnosis of lumbar disc herniation with surgical indication was based on radiological (MRI) evidence of a posterior or posterolateral disc prolapse with compromise to the nerve root or foramina, plus associated congruent symptoms (i.e., radicular pain or sensory/motor deficits in accordance with the level affected radiologically).

Additionally, a control cohort of healthy subjects was recruited from the Orthopaedics Clinic of the same participating institution. These were patients with no lumbar degenerative disease related symptoms. In this cohort, subjects were excluded if: (1) they had previous lumbar surgery, (2) they had asymptomatic vertebral deformities/scoliosis, (3) diagnosis of osteoporosis, vertebral fractures or osteomyelitis.

### 2.3 Preoperative evaluation

All patients were preoperatively evaluated by a senior neurosurgeon specialised in spine disease. Besides basic neurological examination, patients completed a pain assessment with the Visual Analogue Scale (VAS) and a disability assessment according to the Oswestry Low Back Pain Disability Questionnaire (ODI).

In all the cases, an MRI study was conducted including T1, T2 and STIR sequences, in sagittal, coronal and axial planes. This served to establish the diagnosis if lumbar disc herniation, to determine the level affected, and to rule out other causes of compressive radiculopathy. Additionally, all patients underwent lateral standing scoliogram to evaluate sagittal balance.

### 2.4 Surgical technique

Lumbar microdiscectomy was performed with the patient in a modified knee–chest position, under spinal anaesthesia and conscious sedation. The surgical technique was systematic in all cases and included midline incision of 3–4 cm, hemilaminotomy with diamond drill and rongeurs, partial flavectomy and epidural plane dissection to identify the descending nerve root and the prolapsed disc. Extraction of the herniated disc with microlumbar discectomy forceps and curettes, until an adequate root decompression was achieved.

### 2.5 Postoperative evaluation and follow-up

Patients were evaluated 1, 3, 12 and 24 months after the procedure. This evaluation included neurological examination, and measurements of lumbar and radicular pain (VAS) and disability (ODI). A control scoliogram was performed after 12 and 24 months.

### 2.6 Imaging analysis and sagittal balance measurements

Imaging evaluation included complete spinal radiographies (scoliogram) in antero-posterior and lateral positions. These were standardised in “clavicle position” and included from C7 to the femoral heads (8). Images were evaluated by a single radiologist with experience in spinal imaging assessment. Two measurements were performed by this single observer, spared at least two weeks, to minimise the variability. For final analysis, the mean value of the two measurements was taken as a reference.

The quantitative assessment of lumbar biomechanics included the following parameters (Supplementary Table S1):

- Lumbar Lordosis (LL), angulation between the superior endplate of L1 and the superior endplate of S1.
- Thoracic Kyphosis (TK), angulation between the superior endplate of T4 and the inferior endplate of T12.
- Sagittal Balance (sagittal vertical axis, SVA), perpendicular distance of a plumb line going from C7 (C7PL) to the posterosuperior corner of the sacral body. It is considered negative when C7PL was posterior to the posterosuperior sacral point. A normal sagittal balance was defined as a SVA <5 mm and an abnormal (sagittal disbalance) was define as a SVA >5 mm (9).

The pelvic parameters included:

- Pelvic Incidence (PI), angulation between a perpendicular line passing through the midpoint of the sacral plate and a line connecting this point with the femoral head axis (i.e., mean point of the two femoral heads in the lateral projection).
- Sacral Slope (SS), angulation between the sacral plate and the horizontal line.
- Pelvic Tilt (PT), angulation between the vertical line and the line connecting the medial sacral point with the hip axis. It is considered positive when the hip axis is situated in front of the medial sacral point.

### 2.7 Statistical analysis

Statistical analysis was performed Statistical Package for the Social Sciences (SPSS) version 21.0 (IBM). Continuous variables were expressed as mean and standard deviation. Qualitative variables were expressed as percentages. Group differences were assessed with Student t test or Mann–Whitney, as appropriate after normality evaluation with Kolmogorov test. The null hypothesis was that both interventions were equal and a *p*-value of <0.05 (two tailed) was considered an indicator of statistical significance.

## 3 Results

### 3.1 Baseline characteristics of the sample

The study included 52 patients treated for lumbar discectomy. In this cohort, 27 (51.9%) were male, with a mean age of 47.6 (SD 8.8) years, mean weight of 76.0 (SD 16.4) kg, mean size of 1.7 (SD 0.1) m, and mean body mass index (BMI) of 25.5 (SD 4.8).

The control group included 45 healthy subjects without lumbar symptoms. Here, 43 (95.5%) were male, mean age of 33.9 (SD 7.8), mean weight and height of 74.4 (SD 7.9) kg and 1.8 (SD 0.6) m and mean BMI of 23.6 (SD 1.6) (Table 1).

**Table 1:** Demographic and antropometric characteristics of the case-control cohorts.

Variable	Healthy controls (*n* = 45)	Discectomy cases (*n* = 52)	*p*-value
Age. mean (SD)	33.98 (7.81)	47.62 (8.79)	<0.001
Sex (male), *n* (%)	43 (95.56%)	27 (52.94%)	0.002
Weight (kg), mean (SD)	74.04 (7.98)	76.04 (16.42)	0.443
Height (cm), mean (SD)	177 (6)	171 (11)	0.003
BMI, mean (SD)	23.60 (1.63)	25.52 (4.79)	0.025

### 3.2 Outcomes after surgical treatment

**Figure 1:**
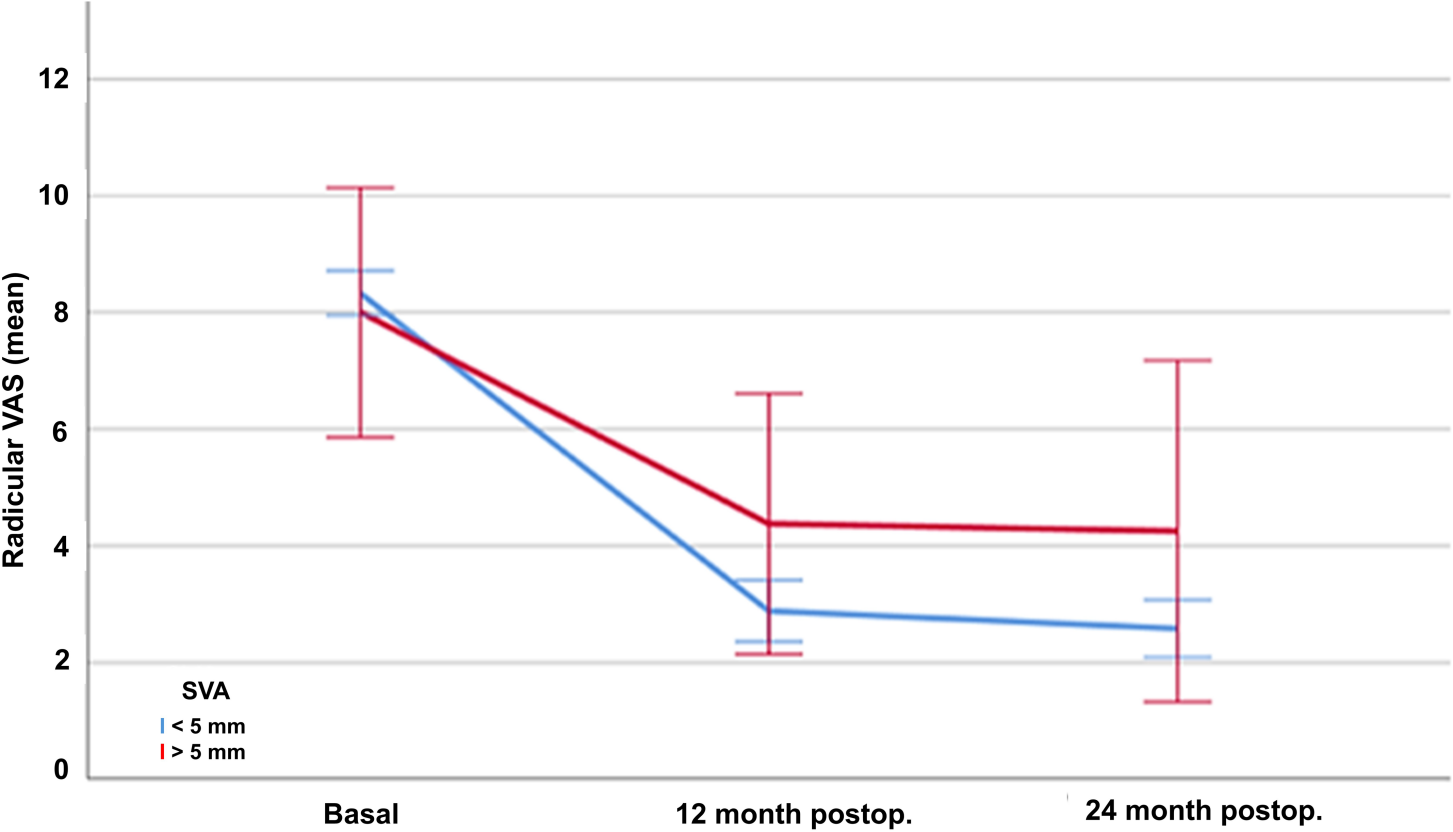
Evolution of mean disability scores according to the Oswestry Low Back Pain Disability Questionnaire (ODI) in patients treated with lumbar discectomy, at baseline and one and twelve months after surgery.

**Figure 2:**
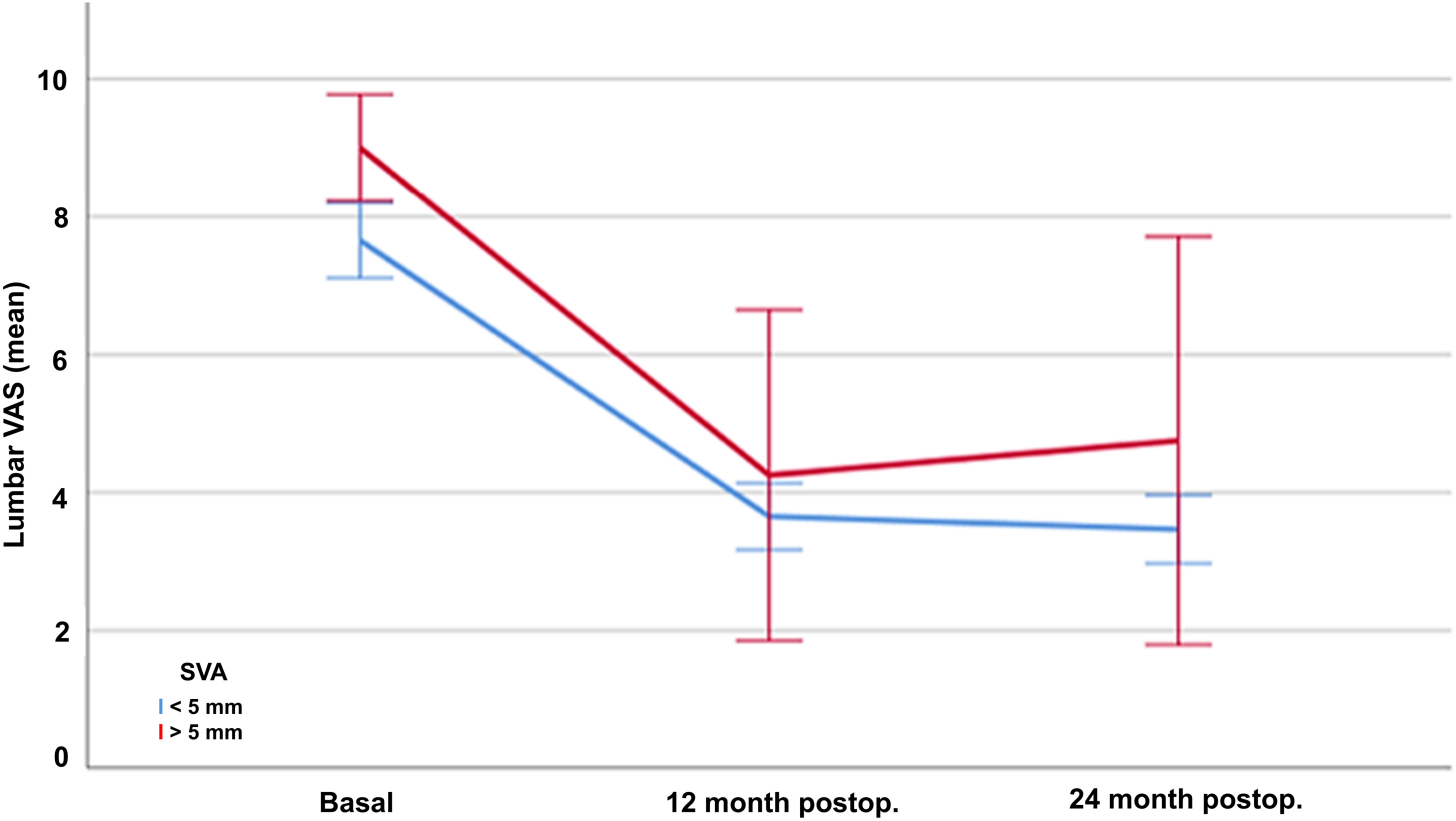
Evolution of radicular pain according to the Visual Analogue Scale (VAS) in patients treated with lumbar discectomy, at baseline and one and twelve months after surgery.

**Figure 3:**
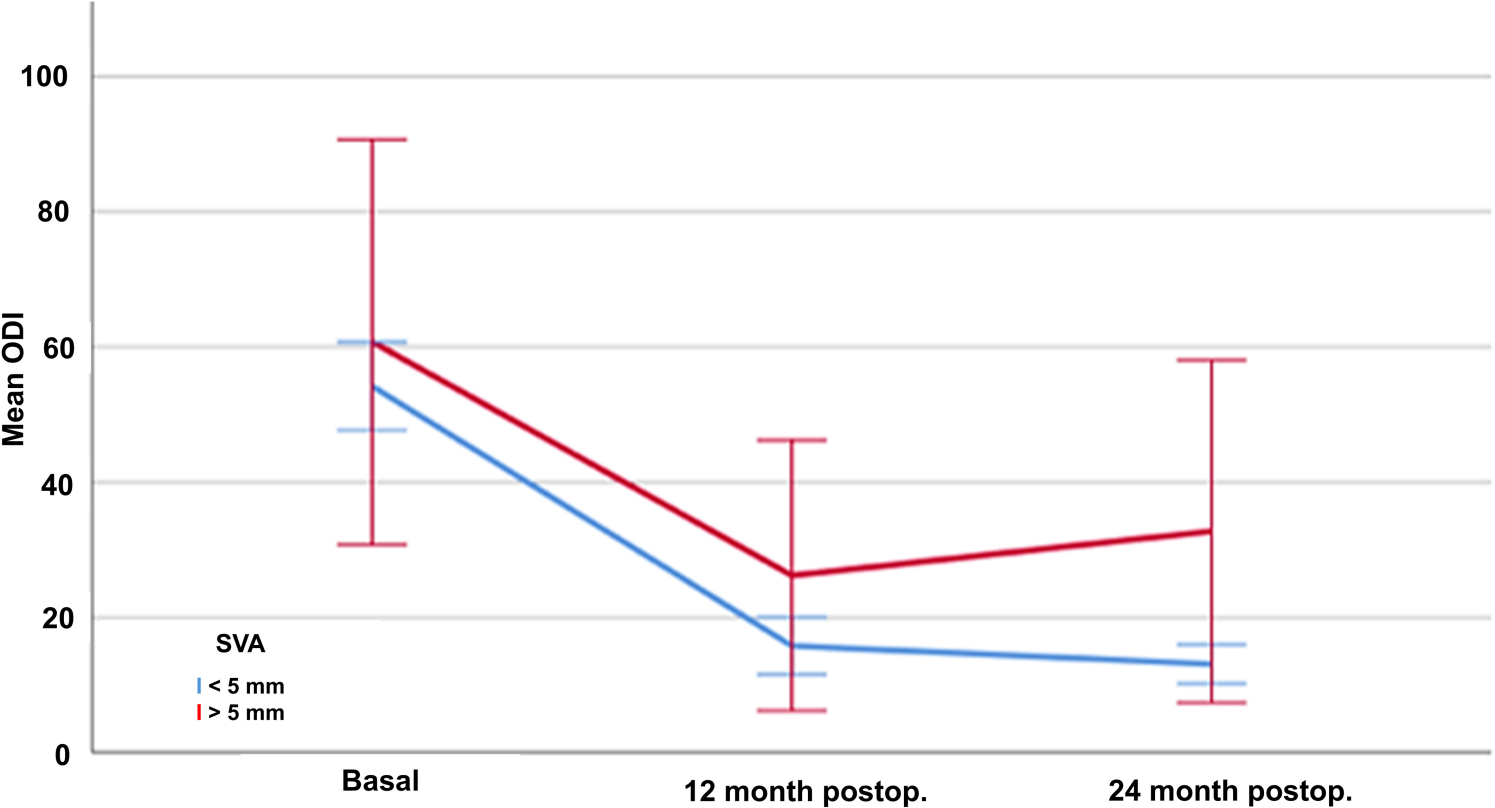
Evolution of lumbar pain according to the Visual Analogue Scale (VAS) in patients treated with lumbar discectomy, at baseline and one and twelve months after surgery.

Basal (presurgical) assessment of pain and disability are shown in Table 2. Postoperative results showed a significant improvement of the three parameters over the two-year of follow-up. The mean absolute reduction of disability according to the ODI was 39.14 (70%) at one year and 40.17 (71%) after two years (*p* < 0.001) (Figure 1). Radicular pain also decreased by a mean of 5.29 (64%) points in the VAS at one year and 5.48 (66%) points after two years (*p* < 0.001) (Figure 2). Lumbar pain also improved a mean of 4.11 (52%) points at one year and 4.27 (54%) points in the VAS after two years (*p* < 0.001) (Figure 3) (Table 2).

**Table 2:** Functional and pain outcomes after lumbar microdiscectomy in the case cohort.

	Baseline	After surgery 12 m	Absolute reduction (%) 12 m	*p*-value	After surgery 24 m	Absolute reduction (%) 24 m	*p*-value
ODI	56.22 (23.28)	17.08 (16.22)	39.14 (70%)	<.001	16.05 (15.72)	40.17 (71%)	<.001
Radicular VAS	8.32 (1.43)	3.03 (1.99)	5.29 (64%)	<.001	2.84 (2.10)	5.48 (66%)	<.001
Lumbar VAS	7.89 (1.78)	3.78 (1.84)	4.11 (52%)	<.001	3.62 (2.06)	4.27 (54%)	<.001

Values are given in mean (SD) unless otherwise specified.

There were 3 cases of recurrence in the form of a prolapsed disc in the same location (i.e., level and side) as the one initially operated. This small number of cases did not allow to perform a statistical comparison of the biomechanical metrics between recurrent and non-recurrent cases.

### 3.3 Baseline radiological evaluation of spinal biomechanics

**Figure 4:**
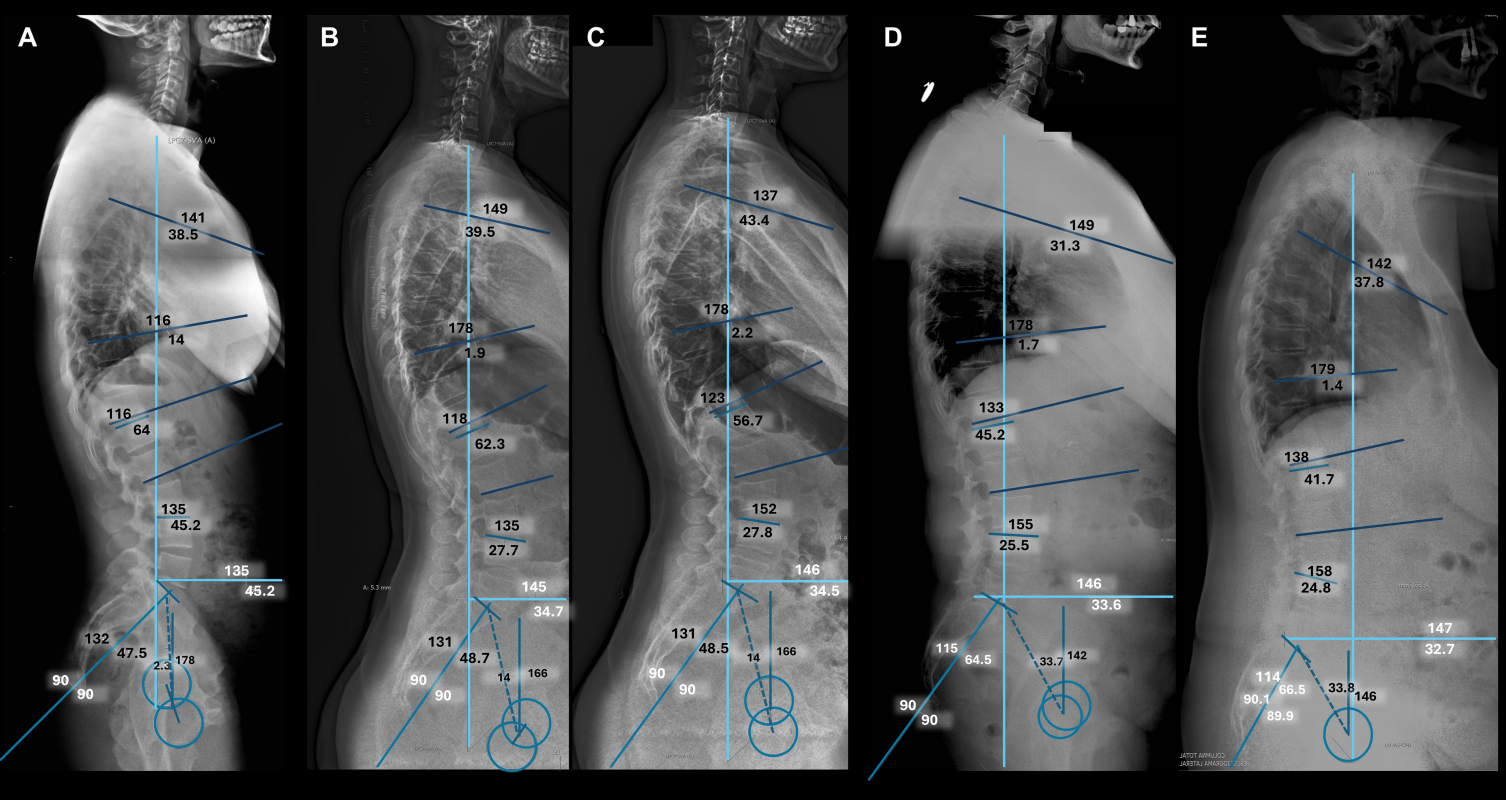
Illustrative cases. Complete spinal radiographies (scoliogram) in lateral projection. **(A)** Normal control in which a neutral sagittal balance is demonstrated. **(B)** Preoperative and **(C)** Postoperative study of a patient with a prolapsed lumbar disc at the level of L5-S1, in which a normal sagittal balance has been demonstrated before and after microdiscectomy. **(D)** Preoperative and **(E)** Postoperative study of a patient with a normal sagittal balance before surgery, and an abnormal sagittal balance after surgery.

A comparative analysis of spinal biomechanical parameters between healthy controls and the group of patients with symptomatic lumbar disc herniation is shown in Table 3. Some illustrative examples are shown in Figure 4. Several differences were observed. First, the TK angulation was more pronounced in in healthy controls (39.03° SD10.17) than in lumbar patients (34.42 SD10.79) (*p* = 0.034). The angulation in the thoraco-lumbar transition (T10-L2) was also more pronounced in the healthy control (6.79° SD7.21 vs. 2.08° SD8.5, *p* = 0.005), and so was the LL (59.54° SD11.19 vs. 48.36 SD7.79, *p* < 0.001) and the lumbo-sacral angulation L4-S1 (40.20 SD13.14 vs. 29.16° SD 5.59, *p* < 0.001). Pelvic parameters also showed significant differences between healthy subjects and lumbar disc patients. The mean PI was 54.71° (SD10.95) in controls and 49.86° (SD7.80) in cases (*p* = 0.014); while the SS was 42.07° (SD8.72) and 33.34° (SD 5.87) respectively (*p* < 0.001).

**Table 3:** Baseline biomechanic variables of the spine of the case-control cohorts.

Parameters	Healthy controls (*n* = 45)	Discectomy cases (*n* = 52)	*p*-value
TK (T4-T12)	39.03 (10.17)	34.42 (10.79)	**0** **.** **034**
T10-L2	6.79 (7.21)	2.08 (8.50)	**0** **.** **005**
LL (L1-S1)	59.54 (11.19)	48.36 (7.79)	**<0** **.** **001**
LLL (L4-S1)	40.20 (7.60)	29.28 (5.59)	**<0** **.** **001**
LDI (LLL/LL)	68.43 (13.23)	61.08 (11.00)	**0** **.** **004**
PI = SS + PT	54.71 (10.95)	49.86 (7.82)	**0** **.** **014**
SS	42.07 (8.72)	33.34 (6.02)	**<0** **.** **001**
PT	12.64 (6.33)	16.52 (6.07)	**0** **.** **003**
SVA mm	−3.09 (29.21)	15.04 (38.92)	**0** **.** **013**

Values are given in mean (SD). Statistically significant differences (*p* < 0.05) are shown in bold.

Of note, the sagittal balance (SVA) had a tendency towards negative values in the healthy group −3.09 mm (SD 29.21) and towards positive values in the lumbar disc group 15.04 mm (SD38.92) (*p* = 0.013).

### 3.4 Correlation of sagittal balance and postoperative outcomes

There were no significant differences in the disability values (ODI) between patients with normal and abnormal sagittal balance preoperatively (ODI 54.58 SD21.40 vs. ODI 64.67 SD 32.46, *p* = 0.426). Noteworthy, the disability outcomes 12 months after surgery were significantly different according to the sagittal balance at baseline. Indeed, patients with normal sagittal balance had better disability outcomes (12mo postoperative ODI 14.52 SD12.81) than those with sagittal disbalance (30.33 SD25.69) (*p* < 0.001). This effect was maintained 24 months after surgery (ODI 12.71 SD8.71 vs. 29.92 SD 35.60 respectively, *p* < 0.001).

Baseline radicular pain according to the VAS were similar regardless of the sagittal balance (8.32 SD1.28 in patients with normal balance, and 8.33 SD2.25 in those with disbalance, *p* = 0.990). Similarly to the disability outcomes, radicular pain improved significantly more in patients with normal sagittal balance both 12 (VAS 2.74 SD1.63 vs. 5.58 SD4.94, *p* < 0.001) and 24 (2.55 SD 1.59 vs. 4.77 SD5.15, *p* < 0.001) months after surgery.

Lumbar pain was significantly different according to the sagittal balance even at baseline (VAS 7.68 SD1.83 with normal balance vs. 9.00 SD0.89 with sagittal disbalance, *p* = 0.004). After surgery, the decrease in lumbar pain was more evident in those with normal sagittal balance, both after 12 (VAS 3.61 SD1.54 vs. 4.06 SD3.28, *p* < 0.001) and 24 (VAS 3.39 SD1.63 vs. 4.29 SD3.47, *p* < 0.001) months from the lumbar discectomy (Table 4).

**Table 4:** Surgical outcomes according to the sagittal balance.

		Time point	Normal sagittal balance	Abnormal sagittal balance	Difference (CI 95%)	*p*-value
ODI	ODI	Basal	54.58 (21.40)	64.67 (32.46)	−10.09 (−34.93)	0.426
		12 months	14.52 (12.81)	30.33 (25.69)	−15.81 (−35.10)	<0.001
		24 months	12.71 (8.71)	29.92 (35.60)	−17.21 (−42.69)	<0.001
Radicular VAS	Radicular VAS	Basal	8.32 (1.28)	8.03 (2.25)	−0.01 (−1.71)	0.990
		12 months	2.74 (1.63)	5.58 (4.94)	−2.84 (−4.08)	<0.001
		24 months	2.55 (1.59)	4.77 (5.15)	−3.22 (−4.52)	<0.001
Lumbar VAS	Lumbar VAS	Basal	7.68 (1.83)	9.00 (0.89)	−1.32 (−2.23)	0.004
		12 months	3.61 (1.54)	4.06 (3.28)	−0.45 (−3.32)	<0.001
		24 months	3.39 (1.63)	4.29 (3.47)	−0.9 (−4.10)	<0.001

Values are given in mean (SD).

## 4 Discussion

In this prospective analytic study, we have demonstrated that lumbar spine biomechanics, and concretely sagittal balance variations, affect both the development of lumbar disc disease and its postoperative course after discectomy. It is remarkable that decreased thoracic and lumbar spinal curvatures seem to predispose individuals to the development of symptomatic lumbar disc herniation. Moreover, sagittal disbalance seems to limit the benefit obtained after lumbar discectomy. These observations could lead to important implications in the selection of patients for simple discectomy surgery.

Degenerative lumbar spinal disease is the leading cause of temporal or permanent disability in young and middle-aged adults, causing a major social and economic burden (10, 11). The role of surgical treatment comes from an adequate patient selection and a correct indication of the surgical technique, after an individual case analysis of symptoms and radiological findings. Globally, our results translate a clinical and statistically significant reduction in the radicular and lumbar pain scores (mean reduction of 5.39 and 5.49 points in the VAS, respectively), as well as disability perception (mean reduction in the ODI of 40 points), of patients undergoing lumbar discectomy maintained after 12 and 24 months. Our results are in accordance with the recent literature in terms of radicular and lumbar pain relief (12–15). However, refractory and/or persistent pain after lumbar discectomy is still reported in about 28% of cases (16). This shows that, while most well-selected cases may benefit from this simple procedure, there is still a subgroup of patients in which the microdiscectomy may not suffice. An objective evaluation of the sagittal balance may help in the prediction of surgical failure and ultimately aid in surgical candidate optimization.

Prior studies analysing spinal biomechanics in patients with lumbar disc herniation showed conflicting results (17, 18). While the loss lumbar lordosis and the decrease in the sacral slope seems to be associated with the development of lumbar disc herniation (18), it is probable that this is an structural deformity but rather an acquired one secondary to the loss of height of the segmental affected vertebral level and to compensatory mechanisms to avoid posterior disc hypertension and foraminal stenosis (2). The observed changes could also be due to a rotation of the pelvis in the coxofemoral joint axis, as a compensatory mechanism due to the contraction of extensor muscles of the hip (2). Ragnics et al. and Endo et al. reported a common spino-pelvic alignment pattern in patients with lumbar disc disease. This was characterized by a low pelvic incidence, a low lumbar lordosis, and an anterior translation of the C7 plumb line (17, 18). In our study, the comparison with the healthy control group also revealed that patients with lumbar disc herniation had a lower pelvic incidence, and a lower lumbar lordosis and lumbo-sacral angulation. However, the noted differences could either be a predisposing factor for disc degeneration or a consequence of altered axial load distribution after the disc prolapse (19). Solving this longstanding dilemma is virtually complex, as it would require a longitudinal population-based study with decades of follow-up and a radiation exposure without medical indications.

In terms of sagittal balance, we found that SVA differed between cases and controls. In the latter, the mean values were close to −3 mm, while in the disc herniation patients it was close to +12 mm. The sagittal balance also seemed to influence the surgical outcomes after lumbar discectomy. 12 and 24 months after the intervention, SVA values tended to decrease, but remained at positive values; meanwhile, the global and segmental lumbar lordosis values remained stable, in contrast to some published series (20). Our findings lead to the hypothesis that the observed morphologic differences were not exclusively explained by pain compensatory mechanisms, but rather to a combination of these and the inherent anatomy of the spine in these patients (21).

Our results were remarkable in terms of the prognostic value of the sagittal balance after lumbar discectomy. In general terms, patients with sagittal disbalance preoperatively had worse outcomes than those with normal sagittal alignment in all the evaluated parameters (lumbar and radicular pain). Considering a score of VAS ≤4 as mild, patients with sagittal disbalance maintained a VAS over 4 one and two years after surgery. Moreover, the disability perception after surgery showed less improvement in patients with sagittal disbalance. In fact, they remained with moderate disability scores, compared to mild levels in those with normal sagittal alignment. These observations suggest that the use of the sagittal balance metrics could be useful in the selection of patients for surgery, in the choice of surgical approach (simple discectomy and/or additional arthrodesis) and in the process of informing the patient about the prognosis of his/her spinal disease (22, 23).

As for limitations, the restrictive selection criteria may hamper a wider generalisation of the conclusions hereby obtained. The healthy control group was comprised of healthy individuals under fit conditions, which may be subjected to selection bias. Additionally, the surgical cohort may also have been treated with non-surgical therapies, which could have influenced the overall outcomes. Similarly, time of evolution of the pain symptoms was not available, and this could also have an influence on the postoperative outcomes. Finally, the small number of cases with recurrence in our cohort did not allow to address a comparison of the biomechanical metrics between recurrent and non-recurrent cases; this topic, however, deserves further investigation in larger cohorts. Likewise, management algorithms based on the objective estimations of spine biomechanics in patients with lumbar disc herniation warrants exploration (23).

## 5 Conclusions

Lumbar degenerative disc disease represents a major burden for healthcare systems; thus, its management is determinant. Lumbar discectomy shows overall positive results, with a significant reduction of pain and disability in most cases. However, a subgroup of patients, still not well defined, may experience persistent pain after the intervention. The use of objective measurement parameters of spine biomechanics, as supported by our study, may help identify these patients for which simple discectomy may not suffice, something that could contribute to treatment planification.

## Data Availability

The raw data supporting the conclusions of this article will be made available by the authors, without undue reservation.
