# Supplementary material for: Influence of spine biomechanics and sagittal balance on the outcome of lumbar discectomy
Source: Front Surg. 2025 Feb 12;12:1494780. doi: 10.3389/fsurg.2025.1494780 (PMC11861076; doi:10.3389/fsurg.2025.1494780)
Supplement: Supplementary file 1 [file Table1.docx]

Supplementary Material

# Supplementary Figures and Tables

**Supplementary Table S1.** Definition of parameters of spinal biomechanics.

| **Parameter** | **Abbreviation** | **Description** |
| --- | --- | --- |
| C7 plumb line- sagittal vertical axis (cm) | C7PL-SVA | Horizontal distance between the posterior corner of the sacrum and the C7 plumb line^a^ |
| Trunk shift (cm) | Trunk shift | Horizontal distance between the C7 plumb line and the Center Sacral Vertical Line^b^ |
| Thoracic kyphosis (°) | TK | Cobb angle method between T4 and T12 |
| Lumbar lordosis (°) | LL | Cobb angle method between L1 and S1 |
| Low lumbar lordosis (°) |  | Cobb angle method between L4 and S1 |
| Pelvic inciSDnce (°) | PI | Angle between the perpendicular line from the sacral plate and the line connecting the midpoint of the sacral plate to the bicoxofemoral axis |
| Pelvic tilt (°) | PT | Angle between the line connecting the midpoint of the sacral plate to the bicoxofemoral axis and the vertical plane |
| Sacral slope (°) | SS | slope angle between superior endplate of S1 and horizontal line |
